# Brief transdiagnostic cognitive behavioral therapy and expressive writing for hazardous drinking and posttraumatic stress symptoms among sexual and gender minority adults: Pilot trial protocol

**DOI:** 10.1016/j.conctc.2026.101666

**Published:** 2026-06-27

**Authors:** Virinca Jaipuriyar, Kriti Behari, Katya Rashkovsky, Rachel Girard, Anna L. Sherman, Natalie Chasten, Gabriella (Gabby) Epshteyn, Joonwoo Lee, Emily C. Helminen, Jillian R. Scheer

**Affiliations:** a Department of Applied Psychology, Graduate School of Applied and Professional Psychology, Rutgers, the State University of New Jersey, Piscataway, NJ, USA; b Department of Psychology, Syracuse University, Syracuse, NY, USA; c Department of Psychology, University of Rhode Island, Kingston, RI, USA; d Department of Counseling, University of Wisconsin-Madison, Madison, WI, USA; e Brown University, Center for Alcohol and Addiction Studies, Providence, RI, USA

**Keywords:** Telehealth, Sexual minority, Gender minority, Expressive writing, Unified protocol, Posttraumatic stress, Hazardous drinking

## Abstract

**Background:**

Sexual minority cisgender women, transgender men and women, and nonbinary or gender diverse (SMW/TGD) people experience disproportionately high rates of hazardous drinking and posttraumatic stress symptoms, due in part to elevated exposure to trauma, stigma-related stressors and related adaptations, and barriers to accessing affirming care. Existing integrated alcohol–trauma interventions have rarely been evaluated or culturally adapted for SMW/TGD people, underscoring the need for scalable, culturally responsive approaches for this population. This protocol describes development and pilot testing of Recovery through Inhibitory learning, Self-Efficacy promoting, problem-solving, and community building (RISE), a brief, culturally responsive telehealth intervention integrating selected Unified Protocol modules with exposure-based expressive writing for trauma-exposed SMW/TGD adults with hazardous drinking and at least subthreshold PTSD symptoms.

**Methods:**

Guided by the ADAPT-ITT framework, Aim 1 will adapt RISE using community- and provider-engaged methods with 20 SMW/TGD community members and 10 mental/behavioral health providers. Aim 2 will pilot RISE in a randomized trial comparing immediate treatment (*n* = 30) versus 6-week waitlist control (*n* = 30). RISE includes four adapted Unified Protocol sessions followed by five days of expressive writing about trauma, stigma, or other adverse experiences with clinician check-ins. Feasibility, acceptability, implementation indicators, and preliminary clinical outcomes will be assessed through self-report and clinician-administered measures.

**Conclusion:**

This study will generate preliminary data regarding feasibility, acceptability, and clinical promise of a brief, scalable intervention addressing hazardous drinking, posttraumatic stress, and stigma-related stressors and related adaptations among SMW/TGD populations. Findings will inform refinement of RISE and a future fully powered randomized controlled trial.

**Trial registration number:**

Registered on 15 October 2025 (ClinicalTrials.gov identifier: NCT07217795)

Sexual minority cisgender women, transgender men and women, and nonbinary or gender diverse (SMW/TGD) individuals face sexual and gender identity-related stigma operating across intrapersonal, interpersonal, and structural levels, which undermines wellbeing and constrains access to opportunities and resources [[1], [2], [3]]. These stressors undermine social safety, increase vulnerability to trauma exposure (e.g., adverse childhood experiences, sexual assault, intimate partner violence) and contribute to maladaptive coping behaviors, such as hazardous drinking, and posttraumatic stress symptoms [[4], [5], [6], [7], [8], [9], [10], [11], [12]]. Reflecting these intersecting adversities, SMW/TGD people report higher prevalence and severity of hazardous drinking and posttraumatic stress symptoms than cisgender heterosexual individuals and sexual minority cisgender men [5,[13], [14], [15]]. These conditions frequently co-occur, contributing to functional impairment, poorer treatment outcomes, elevated suicide risk, and increased mortality [[16], [17], [18], [19], [20], [21], [22]].

SMW/TGD people also face substantial barriers to care, including discriminatory healthcare policies, limited affirming providers, scarce culturally responsive programming, and interventions that rarely attend to intersecting stigma- and trauma-related stressors facing this community [12,23,24]. Few interventions address both hazardous drinking and posttraumatic stress symptoms among SMW/TGD populations. The present project seeks to address this gap by developing and piloting a telehealth intervention integrating transdiagnostic cognitive-behavioral therapy (CBT; i.e., Unified Protocol; [25]) with brief exposure-based expressive writing (EW) to reduce hazardous drinking and posttraumatic stress symptoms among SMW/TGD people.

## 1 Shared mechanisms linking stigma, hazardous drinking, and posttraumatic stress

Hazardous drinking and posttraumatic stress symptoms share overlapping emotional, cognitive, and behavioral mechanisms, supporting the use of integrated, transdiagnostic intervention approaches [[26], [27], [28], [29]]. Core shared processes include negative emotionality [[30], [31], [32], [33]], cognitive biases and psychological inflexibility [34,35], and emotion-driven coping behaviors [[36], [37], [38]], which often emerge within contexts of early and cumulative adversity. In addition, the self-medication model [39] posits that alcohol can be used to regulate distressing affect and posttraumatic stress symptoms [20,40], whereas mutual maintenance models suggest hazardous drinking can perpetuate posttraumatic stress through disrupted emotional processing and extinction learning [38,40,41].

Among SMW/TGD people, these mechanisms operate within broader contexts of stigma, chronic vigilance, and constrained social safety [42]. Stigma-related stressors (e.g., rejection, discrimination) and related adaptations (e.g., identity concealment, internalized stigma) reinforce emotion dysregulation, shame, rumination, and coping-motivated drinking while intensifying posttraumatic stress symptoms [[42], [43], [44], [45], [46], [47], [48], [49]]. SMW/TGD people report not only using alcohol to reduce distress but also to enhance positive affect, belonging, or affirmation that may be difficult to access amid chronic stigma exposure and social unsafety [50,51]. Further, alcohol-centered spaces may provide important opportunities for community connection among SMW/TGD people, although they can also normalize hazardous drinking [52,53]. These environments may expose individuals to harassment and re-traumatization [[52], [53], [54]], while reinforcing patterns of frequent or heavy alcohol use and associated harms among SMW/TGD people [[54], [55], [56]].

### 1.1 Integrated treatment approaches and remaining gaps in SMW/TGD care

Several evidence-based interventions address co-occurring hazardous drinking and posttraumatic stress, including Concurrent Treatment of PTSD and Substance Use Disorders Using Prolonged Exposure (COPE), Seeking Safety, and other CBT-based integrated approaches [40,[57], [58], [59], [60], [61]]. Targeting mechanisms, such as emotion dysregulation, avoidance, trauma processing, and coping-motivated substance use, these interventions improve posttraumatic stress symptom severity, and in some cases, alcohol use outcomes [40,[57], [58], [59], [60], [61]]. Findings also suggest that reducing hazardous drinking may require additional behavioral strategies [57].

Although alcohol- and trauma-focused interventions demonstrate efficacy in predominantly cisgender, heterosexual populations, their effectiveness, relevance, and sensitivity to SMW/TGD people remains unclear. Clinical guidance emphasizes affirmative therapy and culturally responsive care that addresses identity, community, and structural context [62,63]. Yet, sexual orientation and gender identity outcomes are rarely (between 0.4% and 3.8% of the time) examined in alcohol or trauma treatment trials and SMW/TGD-tailored programming remains uncommon [12,62,64]. However, given elevated rates of trauma, sexual and gender identity-related stigma, and universal stressors, SMW/TGD people comprise a large proportion of the population seeking treatment for posttraumatic stress and hazardous drinking [62]. Little is known about whether integrated interventions should be adapted to address early and ongoing trauma exposure, stigma-related stressors, and structural inequities that contribute to SMW/TGD populations’ disproportionate risk of hazardous drinking and posttraumatic stress [[65], [66], [67], [68], [69], [70]].

Early adaptation efforts show promise but highlight persistent gaps. Group-based Seeking Safety demonstrated benefits for hazardous drinking and posttraumatic stress among transgender women living with HIV [65], and interventions tailored to address stigma-related stressors have improved mental health outcomes among sexual and gender minority populations, particularly among those with greater internalized stigma [67,70]. Although several integrated alcohol–trauma interventions demonstrate efficacy in general populations, few have been culturally adapted, evaluated, or designed to concurrently address hazardous drinking, posttraumatic stress, and stigma-related stressors among SMW/TGD populations [71,72]. These gaps suggest the need for integrated, culturally responsive interventions capable of addressing hazardous drinking, posttraumatic stress, and stigma-related distress among SMW/TGD populations [[73], [74], [75], [76], [77], [78]].

### 1.2 Rationale for transdiagnostic CBT

Traditional CBT approaches demonstrate efficacy for hazardous drinking and posttraumatic stress; however, these co-occurring conditions frequently involve overlapping emotional, cognitive, and behavioral mechanisms [[73], [74], [75], [76]]. Among SMW/TGD populations, these processes often unfold alongside stigma-related stressors, chronic threat vigilance, social unsafety, repeated adversity, and complex comorbidity, producing heterogeneous clinical presentations that may not align neatly with disorder-specific treatment models [[27], [28], [29], [30], [31], [32], [33], [34], [35], [36], [37], [38],[42], [43], [44], [45], [46], [47], [48], [49]]. Integrated trauma and substance use treatments (e.g., COPE) are effective but are often lengthy, therapist-intensive, and can involve treatment burden and variable retention, particularly among people with comorbidities [[79], [80], [81], [82], [83]]. These considerations support transdiagnostic approaches that target shared processes (e.g., avoidance, cognitive bias) underlying hazardous drinking, posttraumatic stress, and stigma-related stressors and adaptations [[27], [28], [29], [30], [31], [32], [33], [34], [35], [36], [37], [38],^62^,^75^,^76^,^84^].

The Unified Protocol (UP) is a CBT-based, mechanism-focused, transdiagnostic intervention designed to modify maladaptive responses to emotional experiences through psychoeducation, mindful emotional awareness, cognitive flexibility, and behavioral change [25]. Candidate transdiagnostic mechanisms implicated in hazardous drinking and posttraumatic stress, including negative emotionality, cognitive inflexibility, avoidance, and emotion-driven behaviors, map closely onto the UP framework [[27], [28], [29], [30], [31], [32], [33], [34], [35], [36], [37], [38],^75^,^76^]. These vulnerabilities are particularly relevant among SMW/TGD populations, whose emotional experiences often occur within ongoing contexts of cissexism, heterosexism, and sexism as well as insufficient social safety [[42], [43], [44], [45], [46], [47], [48], [49],^62^]. Compared with disorder-specific protocols, transdiagnostic approaches such as the UP may offer greater flexibility for addressing hazardous drinking, posttraumatic stress symptoms, generalized emotional vulnerability, and stigma exposure (e.g., intersecting heterosexism and sexism) and related adaptations (e.g., anticipated rejection) within a single treatment framework [25,62].

The UP's modular design further supports its suitability for SMW/TGD populations and telehealth intervention development [25,62,75,76]. Rather than requiring delivery of the full 12–16 session protocol, the UP permits flexible sequencing and selection of skills while maintaining fidelity to core treatment principles [75,76]. This flexibility may be especially valuable given accessibility barriers, treatment burden, and heterogeneous clinical presentations common among individuals with co-occurring hazardous drinking and traumatic stress [76]. Evidence also supports reordering and condensing UP modules to target pretreatment skill deficits and shared maintaining mechanisms when full protocol delivery is impractical [25].

This pilot study therefore draws on UP Modules 2–5, which target emotional awareness, cognitive flexibility, maladaptive coping patterns, and emotion-driven behaviors that sustain hazardous drinking, posttraumatic stress, and stigma-related distress [25,48,62,70]. Delivering these core skills early may strengthen coping capacity, improve emotional preparedness, and enhance readiness for subsequent trauma-focused work while maintaining a brief, scalable, and culturally responsive intervention framework. Selected UP modules may help SMW/TGD people identify the emotional and interpersonal functions alcohol serves, increase awareness of emotion-driven drinking patterns, strengthen cognitive and behavioral flexibility, and develop alternative strategies for accessing connection, affirmation, and other valued emotional experiences [85]. Although the UP is not a substance use–specific intervention, it may be particularly relevant for hazardous drinking maintained by affect regulation, avoidance, and interpersonal reinforcement.

### 1.3 Rationale for brief exposure-based expressive writing

Exposure-based interventions are effective approaches for posttraumatic stress because they target avoidance, emotional processing, inhibitory learning, and maladaptive beliefs that maintain posttraumatic symptoms [[79], [80], [81], [82], [83], [84],^86^]. Writing-based exposure approaches, including expressive writing (EW) and Written Exposure Therapy (WET), extend these principles through structured written engagement with stressful or traumatic experiences. Across trauma-exposed populations, writing-based interventions demonstrate benefits for posttraumatic stress symptoms and related mental health outcomes while typically requiring fewer sessions and less therapist time than many traditional trauma-focused treatments [[79], [80], [81], [82], [83], [84],[86], [87], [88], [89], [90], [91], [92], [93], [94], [95]]. These features make writing-based approaches particularly relevant and well-suited for integrated interventions targeting co-occurring hazardous drinking and posttraumatic stress [[96], [97], [98], [99], [100], [101]].

Several considerations informed selection of EW rather than direct adoption of WET. WET employs standardized psychoeducation and repeated writing about a single Criterion A trauma [[79], [80], [81], [82]], whereas this pilot study was designed for the heterogeneous, cumulative, and identity-related adversities commonly experienced among SMW/TGD populations. In addition, rather than relying on a brief introductory session alone as per the WET protocol, this pilot study embeds writing within selected UP modules that cultivate emotional awareness, cognitive flexibility, and coping skills prior to trauma engagement. This approach was informed, in part, by preliminary findings from our exit interviews suggesting that trauma-exposed SMW/TGD participants who completed our pilot EW trial valued greater preparation for stigma- and trauma-focused writing, including psychoeducation, emotion regulation and coping skills, and structured support for navigating avoidance and emotional activation before, during, and after writing sessions.

Writing instructions differ somewhat across protocols. WET emphasizes structured narration of a single trauma memory and may encourage a degree of observational distance to support tolerability and narrative organization [[79], [80], [81], [82]], whereas EW protocols more commonly emphasize emotional disclosure and exploration of thoughts and feelings surrounding stressful experiences. EW additionally permits greater flexibility in writing prompts and targets. Participants may write about experiences contributing most strongly to current posttraumatic symptoms or significant emotional distress, including Criterion A trauma exposure, cumulative victimization, structural stigma, or other identity-related adversity. Although such experiences may not uniformly satisfy *DSM-5* Criterion A definitions, they are often psychologically salient and strongly implicated in posttraumatic stress symptoms and hazardous drinking among SMW/TGD individuals [[42], [43], [44], [45], [46], [47], [48], [49]]. This flexibility may be particularly important for populations whose distress frequently reflects intersecting trauma- and stigma-related experiences.

Other considerations also support the use of EW within this pilot trial. Most WET trials have excluded individuals with substance use concerns or subthreshold posttraumatic stress disorder (PTSD) [80,83,86,87], despite evidence that trauma-exposed people commonly experience clinically significant distress and engage in maladaptive coping behaviors such as hazardous drinking at subthreshold levels of PTSD [88,89]. Moreover, if untreated, subthreshold PTSD can progress to full PTSD, impair functioning, and complicate treatment engagement [88,89]. In addition, WET has not been evaluated among or adapted for SMW/TGD people [82], and no studies, to our knowledge, have modified WET to address the intersection of trauma and stigma-related determinants of posttraumatic stress symptoms and hazardous drinking [77]. While this pilot trial shares features with WET, it differs in its integration of transdiagnostic CBT skills, flexible writing targets, and explicit attention to hazardous drinking and the intersection of trauma exposure and stigma-related stressors commonly experienced among SMW/TGD people.

### 1.4 Why digital EW for SMW/TGD populations

EW may be particularly well suited to telehealth intervention development and SMW/TGD populations. Compared with conventional talk-based trauma treatments, digital EW offers a flexible format that can reduce logistical barriers related to travel, scheduling, provider availability, and geographic access to affirming care [81,[87], [88], [89], [90], [91], [92], [93]]. Writing-based approaches may also afford greater privacy, autonomy, and perceived control over verbal emotional disclosure and pacing, features that may be especially valuable for individuals navigating stigma, disclosure concerns, ambivalence toward trauma-focused treatment, or limited access to culturally responsive providers [[97], [98], [99],^102^]. Consistent with these findings and expert guidelines for treating posttraumatic stress symptoms and other comorbidities [[103], [104], [105]], preliminary qualitative findings from our prior pilot EW trial suggested that trauma-exposed SMW/TGD participants valued the digital format because it enhanced accessibility and flexibility, reduced transportation and scheduling barriers, and allowed engagement in a familiar, private environment that facilitated emotional safety, openness, and honest disclosure during writing. Some participants also reported that the virtual format functioned as an “emotional buffer,” making EW feel less intimidating and easier to approach.

Evidence further suggests that EW may yield stronger mental health and substance-related outcomes when paired with clinician involvement or behavioral adjuncts [[98], [99], [100], [101]]. Accordingly, this pilot trial incorporates brief clinician meetings following writing sessions to provide grounding, safety monitoring, normalization, and support while preserving many of the accessibility and scalability advantages of digital writing-based approaches. Prior work also suggests that SMW/TGD populations value flexible, remotely delivered behavioral interventions that acknowledge minority stress, community context, and identity-related experiences [62,[65], [66], [67], [68], [69], [70]]. These considerations support clinician-facilitated digital EW as a feasible, scalable, and culturally responsive component of an integrated intervention for SMW/TGD populations.

### 1.5 Integrating UP core modules with EW: intervention logic and treatment selection

This pilot intervention combines selected UP modules with EW to target complementary mechanisms underlying hazardous drinking, posttraumatic stress, and stigma-related distress [[106], [107], [108], [109]]. UP Modules 2–5 emphasize emotional understanding, mindful emotional awareness, cognitive flexibility, and countering emotion-driven behaviors—processes commonly implicated in posttraumatic stress symptoms and hazardous drinking [25,85,[110], [111], [112], [113], [114], [115], [116]]. Delivering these skills prior to and during EW is intended to support readiness for trauma engagement and coping capacity, particularly among individuals experiencing chronic stigma exposure, complex comorbidity, and social unsafety [85]. EW serves as a focused exposure-based alternative to traditional UP exposure components by providing a structured and flexible opportunity to repeatedly engage with traumatic, stigma-related, and other highly distressing experiences [77,87,98,117]. Writing permits application of UP skills during processing potentially emotionally activating material while supporting culturally responsive engagement with experiences relevant to SMW/TGD populations [70,77,85,99,101,105].

This sequencing is further informed by phased treatment and emotion regulation frameworks suggesting that development of emotional awareness, coping, and regulatory skills may support trauma engagement among individuals with elevated distress, avoidance, or drinking-to-cope behaviors [105,110,[117], [118], [119], [120], [121]]. Selected UP modules may help participants recognize the emotional and interpersonal functions alcohol serves, increase awareness of emotion-driven drinking patterns, strengthen cognitive and behavioral flexibility, and develop alternative strategies for accessing connection, affirmation, and other valued emotional experiences [56,[60], [61], [62],^110^,^115^]. For individuals with co-occurring hazardous drinking and posttraumatic stress symptoms, strengthening these skills prior to or alongside trauma-focused writing may reduce experiential avoidance and reliance on alcohol as a coping strategy while supporting emotional processing and integration of difficult experiences [61,82,83]. Accordingly, this pilot trial leverages the complementary strengths of transdiagnostic CBT and brief exposure-based writing to provide a brief, scalable, culturally responsive intervention for co-occurring hazardous drinking, posttraumatic stress, and stigma-related distress among SMW/TGD populations. To our knowledge, no interventions have integrated UP modules with culturally adapted EW to concurrently target these intersecting processes.

### 1.6 The current study

This protocol paper details the development and pilot testing of ***R****ecovery through* ***I****nhibitory learning,* ***S****elf-****E****fficacy promoting, problem-solving, and community building* (RISE). RISE is a brief culturally responsive telehealth intervention that integrates the UP's core modules (Modules 2-5) with EW for trauma-exposed SMW/TGD people who report hazardous drinking and meet at least subthreshold posttraumatic stress disorder criteria. In this paper, we (1) describe the development of RISE and its components; (2) outline the plan to evaluate acceptability and feasibility of the proposed treatment; and (3) identify our analytic approach for preliminary outcome signals. Given the pilot design of this protocol, outcome findings will be interpreted as preliminary and will inform a subsequent fully powered trial.

### 1.7 Study aims

The study outlined in this protocol has two primary aims. (1) Aim 1 will seek to adapt an evidence-based UP + EW intervention (i.e., RISE) for trauma-exposed SMW/TGD individuals with hazardous drinking and posttraumatic stress symptoms using community- and provider-engaged methods using the ADAPT-ITT [122] framework. Focus groups and interviews will be held with SMW/TGD community members who report hazardous drinking and posttraumatic stress symptoms (*N* = 20). Interviews will also be held with mental and behavioral health providers serving this population (*N* = 10). (2) Aim 2 will evaluate the feasibility, acceptability, and preliminary outcomes of the adapted telehealth RISE intervention in a pilot randomized trial comparing RISE (*N* = 30) to a waitlist control (*N* = 30) among trauma-exposed SMW/TGD adults who report hazardous drinking and meet at least subthreshold PTSD criteria. Implementation outcomes will include feasibility (recruitment/retention, session completion, adherence) and acceptability (satisfaction, perceived fit, burden). Clinical outcomes will include alcohol use (quantity/frequency, alcohol-related problems) and posttraumatic stress symptom severity (self-report and clinician-administered assessment) at post-treatment. This trial has been reviewed by the Institutional Review Board at the University of Rhode Island and registered with ClinicalTrials.gov (NCT07217795).

## 2 Materials and methods

### 2.1 RISE development and evaluation framework

Adaptation and evaluation of the RISE intervention will follow the ADAPT-ITT [122] framework, an eight-phase model guiding adaptation of evidence-based interventions for new populations and contexts (for more detail, see Table 1). Phases I-III, including literature review and needs assessment, selection of intervention components, and preliminary mapping of treatment targets and mechanisms, were completed prior to the current protocol and informed the intervention rationale described in the Introduction. Building on this foundation, Aim 1 will implement ADAPT-ITT Phases IV–VII through iterative adaptation procedures with SMW/TGD community members and mental and behavioral health providers working with SMW/TGD people, and Aim 2 will implement Phase VIII through pilot testing of the adapted intervention.

**Table 1:** ADAPT-ITT model (Wingood et al., 2008) adaptation process.

Adaptation phase	Task	Study phase	Methods
1. Assessment	Who is the new target population and why is it at risk?	Completed	- Conducted a targeted review on hazardous drinking and posttraumatic stress among SMW/TGD populations to define mechanism targets, dosing, and telehealth delivery constraints
2. Decision	What evidence-based intervention is to be adapted?	Completed	- Reviewed literature and identified the need to adapt a brief, telehealth CBT-EW intervention
3. Administration	What in the original evidence-based intervention needs to be adapted and how?	Completed	- Specified core components (UP Modules 2–5 + 5-day EW) based on mechanism mapping - Predefined adaptation parameters by examining manuals, existing literature, and consulting with the research team
4. Production	How to produce and document adaptation to evidence-based intervention?	Aim 1, Step 1	- Develop Draft v0.1 of the RISE manual that preserves active ingredients and theoretical rationale - Create participant handouts, clinician check-in/safety scripts, and fidelity checklists
5. Topic experts	Who can help to adapt the evidence-based intervention?	Aim 1, Step 2	- Conduct semi-structured interviews with 10 mental/behavioral health providers serving SMW/TGD people reporting hazardous drinking and posttraumatic stress symptoms - Conduct focus groups with 20 SMW/TGD community members who report hazardous drinking and posttraumatic stress symptoms; piloting brief elements (e.g., mindfulness exercise, EW prompt) to evaluate clarity, tone, comprehension, burden, cultural responsiveness, feasibility, and perceived barriers
6. Integration	What is going to be included in the adapted evidence-based intervention to pilot?	Aim 1, Step 3	- Analyze qualitative data using a rapid, framework-guided thematic analysis approach - A preliminary codebook—organized around mechanism fit, cultural fit, feasibility, and safety— will be developed, double-coded on an initial subset, and refined to consensus - Maintain an audit trail and discrepancy log - Build a change matrix (issue → evidence → revision → priority/feasibility rating) - Incorporate revisions until no new high-priority modifications emerge - Document adaptation rationale linking each change to community member and provider input and the mechanism framework - Produce Manual v1.0 (instructions, scripts, timing, participant workbook/handouts, fidelity and competence checklists, clinical protocol for conducting risk assessments) - Finalize trial flow (screening, randomization, assessment schedule, data capture, adverse-event monitoring) and implement data quality checks (attention/fraud flags for online components)
7. Training	Who needs to be trained and how?	Aim 2, Step 1	- Establish procedures and standardized training package for RISE interventionists (telehealth orientation, competency goals, didactics on mechanisms and cultural adaptations for SMW/TGD participants, module-specific demonstrations, standardized role-plays, safety planning), supervision plan (format, frequency, case review cadence), fidelity monitoring (checklists, rating rubrics, sampling plan), and assessor training for clinician-delivered measures
8. Testing	Did adaptation work?	Aim 2, Step 2	- Randomize SMW/TGD participants with hazardous drinking and posttraumatic stress symptoms to RISE or waitlist control; deliver the intervention; collect feasibility/acceptability metrics and preliminary alcohol and traumatic-stress outcomes to inform refinement and power calculations for a definitive trial

***Note.*** SMW/TGD = sexual minority cisgender women, transgender women, transgender men, nonbinary people, and gender-diverse individuals; CBT = cognitive behavioral therapy; RISE = **R**ecovery through **I**nhibitory learning, **S**elf-**E**fficacy promoting, problem-solving, and community building.

#### 2.1.1 Trial design

**Fig. 1:**
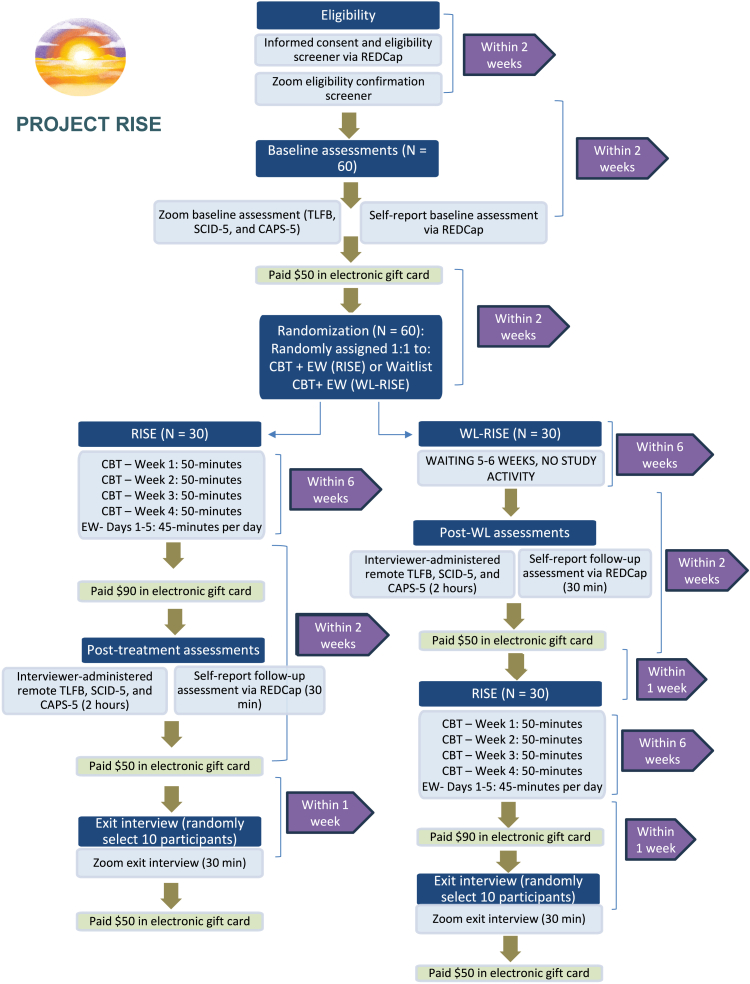
Aim 2 participant flow diagram. Description of the image: The figure depicts study procedures for Aim 2, including eligibility screening, baseline assessment, allocation to condition, RISE intervention/waitlist control, post-treatment assessment, and exit interview. Anticipated schedule, compensation, and measures assessed at each time point are detailed.

To address Aim 2, we will conduct a pilot randomized controlled trial using a parallel, 1:1 unstratified allocation design comparing immediate treatment receipt with a 6-week waitlist control condition (see Fig. 1). Consistent with the pilot nature of the study, treatment outcome findings will be interpreted as preliminary estimates intended to inform feasibility, acceptability, and future efficacy testing. Outcome data will be collected through virtual clinician-administered interviews and online self-report assessments. Participants randomized to immediate treatment will complete assessments at baseline, post-treatment (following completion of UP modules and EW sessions), and 1-week follow-up. Participants randomized to the waitlist condition will complete assessments at baseline, post-waitlist (i.e., second baseline/prior to treatment initiation), post-treatment, and 1-week follow-up (see Fig. 1).

#### 2.1.2 Participants

##### 2.1.2.1 Aim 1: adaptation phase participants

Aim 1 will enroll 20 SMW/TGD community members and 10 mental or behavioral health providers. Eligible community members will: identify as sexual minority cisgender women, transgender women, transgender men, nonbinary people, or gender diverse people (e.g., genderqueer, agender); be ≥ 18 years; speak English; have reliable internet access and access to a smartphone, tablet, or computer; screen positive for hazardous drinking (Alcohol Use Disorders Identification Test-Consumption [AUDIT-C] ≥3 [123]; endorse at least minimal motivation to reduce drinking (Readiness Ruler ≥4) [124]; report ≥1 potentially traumatic event on the Life Events Checklist [125]; and meet at least subthreshold posttraumatic stress disorder criteria on the PTSD Checklist-5 (PCL-5) [126]. Consistent with PTSD assessment recommendations [127], subthreshold PTSD eligibility will require endorsement of ≥1 Criterion B (re-experiencing), C (avoidance), D (negative cognitions or mood), and E symptom (exaggerated arousal). The AUDIT-C cutoff of ≥3 was selected to identify hazardous drinking while maximizing sensitivity to clinically meaningful alcohol-related risk among SMW/TGD populations, recognizing that alcohol-related risk in these communities may occur at comparatively lower levels of alcohol consumption [56]. Cisgender sexual minority men, cisgender heterosexual men, and transgender or gender diverse individuals who identify as heterosexual will not be included given that the intervention is designed to address co-occurring hazardous drinking, posttraumatic stress symptoms, and stigma facing sexual minority cisgender women, transgender women, transgender men, nonbinary people, or gender diverse people. Community members will be required to reside in Rhode Island, Massachusetts, or Connecticut with intent to remain in the area for at least five months to support completion of study activities, including enrollment, intervention participation, post-treatment assessments, and follow-up procedures. This criterion was selected to minimize attrition due to relocation-related issues.

Providers will be eligible if they are ≥18 years old, speak English, currently provide mental or behavioral health services to SMW/TGD clients reporting hazardous drinking and posttraumatic stress symptoms, and either reside in or provide services within Rhode Island, Massachusetts, or Connecticut to ensure familiarity with the regional service environment, referral landscape, and local clinical, sociocultural, and service-delivery contexts relevant to intervention adaptation. For Aim 1, the only exclusion for providers and community members is failure to meet eligibility criteria.

##### 2.1.2.2 Aim 2: pilot randomized trial participants

Aim 2 will enroll 60 SMW/TGD community members randomized 1:1 (unstratified) to immediate RISE or a 6-week waitlist condition. Inclusion criteria will mirror Aim 1 community eligibility criteria. Additional Aim 2 exclusions are intended to reduce treatment contamination and ensure participant safety during this early-stage pilot. Exclusions include: current mental health treatment ≥1 day/month; CBT within the past three months (participants remain eligible to seek care after enrollment); current alcohol or drug use disorder treatment other than mutual self-help (e.g., Alcoholics Anonymous); current PTSD/trauma-focused treatment; need for alcohol detoxification (adapted self-report Clinical Institute Withdrawal Assessment for Alcohol-Revised [CIWA-Ar] ≥15); active psychosis (Revised Behavior and Symptom Identification Scale [BASIS-R] psychosis subscale ≥1); active mania (positive Altman Self-Rating Mania Scale [ASRM] screen); active suicidality (Suicidal Ideation Attributes Scale [SIDAS] ≥22); or legal mandate to attend treatment. Across both aims, community members and providers will be excluded if they fail attention checks or display fraudulent or bot-like responses [128].

#### 2.1.3 Procedures

##### 2.1.3.1 Recruitment and enrollment

Participants will be recruited through flyers, social media, and outreach to local organizations working with sexual and gender minority people, those with lived experience of trauma, or people who use substances, as well as violence-prevention organizations. Interested people will complete an electronic informed consent and eligibility survey, including contact information to schedule a virtual screening with a study team member to confirm eligibility.

**Aim 1 recruitment and enrollment.** Following eligibility confirmation, Aim 1 participants will receive instructions to complete a brief online survey and schedule an interview/focus group. Participation in Aim 1 will conclude following completion of an interview or focus group. Recruitment for Aim 1 began in November 2025 and is projected to conclude June 2026.

**Aim 2 recruitment and enrollment.** Aim 2 recruitment will occur following completion of Aim 1 adaptation activities. Recruitment for Aim 2 will begin after completion of Aim 1 and finalization of the adapted RISE manual and participant workbooks (anticipated August 2026). Following eligibility confirmation, Aim 2 participants will receive instructions to complete a baseline self-report assessment and schedule a virtual clinician-administered baseline assessment prior to randomization.

##### 2.1.3.2 Aim 1 procedures

After eligibility confirmation through the electronic screening survey and synchronous virtual screening, Aim 1 participants will complete a brief (about 10-min) REDCap survey assessing sociodemographic characteristics and relevant treatment experiences (e.g., receiving or providing care related to hazardous drinking, posttraumatic stress, and SMW/TGD populations).

Following survey completion, providers will participate in an individual semi-structured virtual interview examining their experiences delivering treatment to SMW/TGD individuals with hazardous drinking and posttraumatic stress symptoms, perceived treatment gaps, and recommendations for intervention adaptation. Community members will participate in either an individual interview or focus group, according to participant preference, exploring treatment goals, prior treatment experiences, perceived barriers and facilitators to care, and feedback on intervention content and delivery.

Both providers and community members will pilot brief elements of RISE (e.g., psychoeducation excerpts, mindfulness exercise) and provide feedback regarding instructions, clarity, tone, comprehension, burden, cultural responsiveness, feasibility, safety, and perceived fit. Feedback will be synthesized using an adaptation matrix to identify recommended modifications, areas of convergence or divergence across participant groups, and proposed refinements to the RISE manual and participant workbook. Intervention components perceived as unacceptable, unsafe, confusing, excessively burdensome, or insufficiently aligned with treatment targets may be revised, replaced, or removed as appropriate. Proposed adaptations will be evaluated for cultural responsiveness, mechanism alignment, feasibility, and participant safety prior to finalization of intervention materials for Aim 2 pilot testing.

##### 2.1.3.3 Aim 2 procedures

Following eligibility confirmation through the electronic screening survey and synchronous virtual screening, Aim 2 participants will complete online self-report assessments and virtual clinician-administered assessments. Participants randomized to immediate treatment will complete assessments at baseline, post-treatment (following completion of RISE UP modules and EW sessions), and 1-week follow-up. Participants randomized to the 6-week waitlist condition will complete assessments at baseline, post-waitlist/prior to treatment initiation, post-treatment, and 1-week follow-up (see Fig. 1).

Clinician-administered assessments will include the 30-day Timeline Followback (TLFB) [129] to assess alcohol use quantity and frequency; the Structured Clinical Interview for *DSM-5* (SCID-5) Substance Use Disorders module [130] to characterize current and past alcohol/drug use disorders; and the Clinician-Administered PTSD Scale for *DSM-5* (CAPS-5) [131] to determine at least subthreshold PTSD diagnosis and symptom severity. During the baseline virtual visit, participants will schedule all planned intervention sessions and receive a scheduling handout, email reminders for appointments, and clinician contact information for rescheduling if needed. Participants will receive $50 total for completion of baseline self-report and clinician-administered assessments regardless of condition.

**Randomization.** After completion of the baseline self-report and clinician-administered assessments, Aim 2 participants will be randomized 1:1 (unstratified) using REDCap to either immediate treatment receipt or a 6-week waitlist control condition. Randomization assignments will be accessible only to the project coordinator responsible for scheduling participants into the appropriate study timeline. All other study personnel, including assessors, interventionists, and the Principal Investigator, will interact with participants using study IDs and will not be informed of whether participants were assigned to immediate treatment or waitlist. Interventionists and supervisors will know only when a participant is cleared to begin RISE, regardless of whether that occurs immediately after baseline or after the waitlist period. Clinician assessors conducting outcome evaluations will remain masked to condition assignment.

**Immediate condition.** Participants randomized to the immediate condition will begin RISE within 2 weeks following randomization. Participants will be allowed up to 6 weeks to complete the intervention, including four UP-based sessions delivered across five weeks followed by five days of EW completed within 1 week. Participants will also complete post-treatment assessments and a 1-week follow-up assessment. Limited scheduling flexibility (e.g., brief rescheduling, temporary pauses) will be permitted and documented to accommodate participant burden, availability, or clinically indicated needs while maintaining intervention and assessment sequencing.

**Waitlist control condition.** Participants randomized to the waitlist condition will complete baseline assessments identical to those completed by the immediate treatment group and then enter a 6-week wait period. Following the wait period, participants will complete a post-waitlist assessment (i.e., prior-to-treatment assessment) identical to the immediate treatment group's post-treatment assessment. Participants in the waitlist condition will then begin RISE within approximately two weeks of the post-waitlist assessment and will follow the same intervention timeline and assessment schedule as the immediate treatment group, including post-treatment and 1-week follow-up assessments. Providing RISE following the wait period is intended to maintain access to care and support ethical study conduct.

**RISE intervention.** RISE is a brief, telehealth-delivered intervention consisting of four once-weekly adapted UP skill sessions (Modules 2–5) followed by five consecutive days of EW (see Fig. 1). UP sessions will focus on understanding emotions, cultivating mindful emotional awareness, enhancing cognitive flexibility, and countering emotion-driven behaviors in contexts relevant to SMW/TGD people's experiences, such as using alcohol to cope with anticipated stigma, trauma, or difficult emotional states, and relying on avoidance coping to manage distress. Clinicians will be clinical psychology doctoral students trained in the RISE protocol.

Immediately following the four-session skills phase, participants will begin five consecutive days of EW. Participants will receive a secure REDCap link containing the daily writing prompt and a brief follow-up survey at a time jointly selected by the participant and clinician. Five writing days were selected because standard three-session EW protocols may be insufficient for reducing posttraumatic stress symptoms [80]. Participants will be instructed to write privately for 15–20 min about an experience contributing substantially to current traumatic stress, emotional distress, or alcohol-related coping, including a Criterion A trauma identified during the baseline CAPS-5 assessment, stigma-related adversity, or another distressing adverse experience. Consistent with standard EW procedures, participants will focus on their thoughts and feelings rather than grammar, spelling, or structure [83].

Following each writing session, participants will complete a brief (20–25 min) telehealth check-in with the same clinician who delivered their UP sessions. Participants may share their writing through reading aloud, screen sharing, verbal summarization, or may elect not to share the content directly. Clinicians will use process-focused discussion to monitor emotional arousal and safety, reinforce grounding and coping skills, support engagement with difficult material, and connect writing experiences to UP skills such as emotional awareness, cognitive flexibility, and reduction of emotion-driven behaviors. Discussions may include identifying emotional patterns activated during writing, examining inflexible beliefs, and applying coping strategies to manage alcohol cravings or reduce using alcohol to feel better or to cope with distressing trauma reminders, interpersonal rejection, or negative affect.

Clinicians will also provide brief psychoeducation to normalize emotional activation during writing, reinforce the rationale for sustained engagement with difficult memories and emotions, and support understanding of habituation and inhibitory learning [[79], [80], [81], [82], [83], [84],^86^,^132^]. When relevant, clinicians may contextualize identity-linked adversity within broader sociostructural experiences affecting SMW/TGD populations [18,45,85].

This hybrid format preserves privacy during the writing task while adding structured clinician support to promote safety, engagement, and skill generalization. Consistent with inhibitory learning and emotion regulation frameworks [[79], [80], [81], [82], [83], [84],^86^], the intervention pairs repeated engagement with difficult emotional material with coping and regulatory strategies. The massed writing schedule is intended to support sustained emotional processing [80,133].

##### 2.1.3.4 Aim 2 assessment procedures

**Weekly assessments during the UP skills phase**. Participants will complete brief weekly assessments (approximately 15 min) prior to each UP session via REDCap. These assessments are intended to capture weekly changes in symptoms across the skills phase while minimizing immediate influence of session content on participant responses. Measures will assess depression, anxiety, posttraumatic stress symptoms, dissociation, affect, alcohol and other drug use, and alcohol craving. In addition to informing clinical monitoring, these measures will permit exploratory examination of symptom trajectories during treatment. Participants will receive remuneration of $10 for each weekly assessment completed before UP sessions, up to $40 across the UP phase.

**Daily assessments during the EW phase.** Participants will complete brief daily assessments (∼5–7 min)immediately following each writing session and prior to the telehealth clinician check-in. Daily measures will capture proximal, state-level responses to repeated engagement with emotionally salient material, including anxiety/arousal, emotional engagement, dissociation, substance use and craving, and exposure dose indicators (e.g., repeated writing about the same versus different event[s]). Administering assessments before the clinician check-in is intended to characterize immediate responses to the writing task itself, minimize recall bias, support safety monitoring, and permit exploratory examination of inhibitory learning– and emotion regulation–related processes during the writing phase. Elevated distress or dissociation will be reviewed during the same-day clinician check-in and addressed through grounding, safety monitoring, and reinforcement of relevant UP skills. Participants will receive remuneration of $10 for each daily assessment completed following each writing session ($50 total across the EW phase).

**Follow-up assessments.** Participants in the immediate treatment condition will complete follow-up assessments 1 week following intervention completion. Participants in the waitlist condition will complete an equivalent assessment following the 6-week wait period (i.e., prior-to-treatment assessment), as well as post-treatment and 1-week follow-up assessments after completing RISE. Follow-up assessments will include a 30-min online self-report survey and a virtual clinician-administered assessment (∼2 h) consisting of the 30-day TLFB, SCID-5 Substance Use Disorders module, and CAPS-5. Participants will have up to 2 weeks from the target assessment date to complete follow-up procedures and will receive reminder emails. Participants will receive $50 for completing follow-up assessment (either 1-week follow-up following intervention completion for those in the immediate treatment condition or the 1-week assessment following the wait period for those in the waitlist condition).

##### 2.1.3.5 Adherence, fidelity, and contamination procedures

In the immediate arm, adherence will be indexed by UP session attendance (0–4). EW adherence will be indexed by writing completion (0–5), system-captured time-on-task with a minimum writing of ≥15 min, and word count. EW engagement will be characterized using brief post-writing indicators collected immediately following each session, including self-reported prompt adherence, emotional engagement, avoidance, and perceived disclosure.

Prior to data collection, clinician assessors will complete standardized training in study procedures, clinical risk assessment, and administration of clinician-rated measures, including practice administration and reliability calibration. Standardized fidelity and competency checklists will be used to monitor assessment and intervention delivery, with approximately 20% of recorded sessions reviewed by the Principal Investigator and clinical supervisor. Ongoing weekly supervision will support fidelity, reliability, and procedural adherence. Prior to participant contact, interventionists will complete standardized training in the RISE protocol, including UP principles, EW procedures, telehealth delivery, SMW/TGD-affirming care, clinical risk assessment, and study procedures through manual review, didactics, and role-plays.

Participants may discontinue participation at any time without penalty or request scheduling or pacing modifications due to burden, emotional distress, or competing responsibilities. Clinicians will monitor distress, dissociation, alcohol-related risk, suicidality, and other safety concerns during treatment sessions and EW check-ins. When clinically indicated, sessions may be paused, rescheduled, or shifted toward grounding, coping review, safety planning, crisis intervention, or referral to higher levels of care. Initiation of new trauma-focused or alcohol treatment during the study period will be assessed in the post-treatment or post-waitlist assessments. Concomitant treatment will be considered in sensitivity analyses.

Trial safety procedures will include monitoring for serious adverse events, acute suicidality, mania, psychosis, or other exclusionary clinical concerns identified during treatment or clinician-administered assessments. If exclusionary thresholds are met, participation may be discontinued and referrals initiated. The trial may be paused or terminated if continuation is deemed unsafe by the Principal Investigator and/or Institutional Review Board (IRB) based on emerging safety concerns, adverse events, or other clinically significant risk indicators.

##### 2.1.3.6 Acceptability and feasibility

Acceptability and feasibility of RISE will be evaluated across both aims using participant and provider feedback, quantitative implementation indices, and qualitative data.

**Aim 1 pre-pilot adaptation.** SMW/TGD community members and providers will review draft intervention materials and provide feedback regarding clarity, cultural responsiveness, safety, burden, feasibility, and perceived fit with treatment targets and mechanisms. Feedback will be synthesized using an adaptation matrix to identify areas of convergence, divergence, and recommended revisions. This process will inform iterative refinement of intervention materials, procedures, and delivery prior to Aim 2 pilot testing.

**Aim 2 pilot testing.** Treatment feasibility will be operationalized using intervention uptake, completion, retention, implementation burden, and data completeness metrics. Intervention completion will be defined *a priori* as completion of ≥3 of 4 UP sessions and ≥3 EW sessions, consistent with existing recommendations [101,115]. Retention will be indexed by completion of post-treatment and follow-up assessments. Additional feasibility indicators will include recruitment yield, screening-to-enrollment ratios, assessment completion rates, prompt compliance, missing data, staff effort, and frequency of risk assessments or safety escalations.

Acceptability will be assessed at post-treatment and follow-up using standardized measures, including the Client Satisfaction Questionnaire–8 (CSQ-8), Working Alliance Inventory–Short Form (administered following the Unified Protocol phase), Reactions to Research Participation Questionnaire–Revised (RRPQ-R), and a single-item recommendation rating. Participants will also provide brief open-ended feedback regarding study participation.

To deepen understanding of intervention fit, burden, and implementation barriers/facilitators, semi-structured qualitative exit interviews will be conducted with 20 participants selected across study conditions and treatment completion patterns. Immediate-treatment participants will be interviewed following completion of post-treatment procedures, and waitlist participants will be interviewed following crossover completion of RISE. Participants who discontinue or do not initiate or complete treatment will remain eligible for interviews, when feasible, to inform understanding of barriers to engagement and intervention refinements. Interviews and focus groups will be audio-recorded and transcribed verbatim. Transcripts will be deidentified and analyzed using rapid, framework-guided thematic methods to generate refinements [134].

##### 2.1.3.7 Data management and quality assurance

Self-report, EW, and study process measures will be administered via REDCap and stored on secure, university-approved servers using predefined validation procedures (e.g., range checks, required fields, missing-data flags). EW completion and time-on-task metrics will be automatically captured, and selected writing characteristics (e.g., prompt adherence, engagement indicators) will be coded for analytic purposes.

Data quality procedures will include automated screening for fraudulent or bot-like responding (e.g., attention checks, response-pattern review), routine audits of data completeness and consistency, and double-coding of qualitative transcripts. Detailed data management, coding, and quality assurance procedures will be maintained in a separate Data Management Manual.

Participant information will be stored in password-protected, encrypted systems and accessible only to authorized study personnel responsible for recruitment, scheduling, follow-up, and safety monitoring. Audio recordings will be stored separately from identifying information, and deidentified datasets will be generated for analysis. Data will be retained and destroyed according to IRB-approved procedures. Given the minimal-risk nature of the protocol, brief intervention duration, and clinical monitoring, a Data Monitoring Committee is not planned.

#### 2.1.4 Power analysis

Qualitative sample sizes were selected based on established rapid, framework-guided thematic saturation and feasibility. For Aim 1, 20 SMW/TGD community members distributed across several interviews or focus groups are expected to provide sufficient diversity of perspectives regarding hazardous drinking, posttraumatic stress, treatment experiences, and barriers and facilitators to care [134,135]. For provider interviews, empirical studies suggest thematic saturation commonly occurs within focused samples of approximately 8–12 interviews [136]; therefore, a target of 10 providers is expected to be adequate for eliciting perspectives on intervention feasibility, implementation barriers, and best practices in SMW/TGD-affirming care. For Aim 2, recent methodological guidance discourages using pilot trials to establish efficacy or generate definitive effect size estimates [137]. Accordingly, this pilot trial is designed primarily to estimate feasibility, acceptability, recruitment, retention, adherence, and assessment parameters to inform a future fully powered randomized controlled trial. Consistent with recommendations for behavioral pilot trials [138], we will target enrollment of 60 participants randomized across two conditions (30 per condition). The target sample incorporates anticipated attrition of approximately 20%, based on this study team's prior EW intervention research with a similar population [77] and existing telehealth-delivered CBT and EW studies involving SMW/TGD populations [70,99], while recognizing that attrition may be elevated among populations experiencing substantial psychosocial burden and barriers to care. This sample size is expected to provide sufficiently precise estimates of feasibility and acceptability indicators while supporting preliminary examination of clinical outcome trends.

#### 2.1.5 Analytic plan

##### 2.1.5.1 Aim 1

Aim 1 qualitative data will be analyzed using a rapid, framework-guided thematic analysis [134,139] informed by ADAPT-ITT domains (e.g., content, format, delivery, safety), treatment targets, and SMW/TGD-specific considerations relevant to intervention adaptation. An initial codebook will be developed deductively from ADAPT-ITT domains, intervention mechanisms, and SMW/TGD-relevant contextual factors, with iterative refinement through team discussion and memoing. Two coders will independently code an initial subset of transcripts to establish coding consistency and refine codes; subsequent coding will include periodic double-coding, consensus meetings, and adjudication procedures to resolve discrepancies.

Data will be synthesized using a framework matrix approach to identify key themes, convergent and divergent perspectives across community members and providers, and implications for intervention refinement [140]. Findings will be translated into an adaptation matrix documenting proposed modifications, supporting evidence, rationale, feasibility considerations, and priority ratings to guide revisions to the RISE manual and study procedures.

To enhance rigor and transparency, the research team will maintain an audit trail of coding and adaptation decisions, as well as versioned intervention updates. Reporting will follow established standards (e.g., consolidated criteria for reporting qualitative research; [141]).

##### 2.1.5.2 Aim 2

**Acceptability and Feasibility Analyses.** Qualitative feedback regarding intervention acceptability will undergo rapid, framework-guided thematic analysis using coding, consensus, and synthesis procedures consistent with the Aim 1 analytic approach. Quantitative feasibility and acceptability indicators will be summarized descriptively, including recruitment yield, screening-to-enrollment ratios, intervention uptake and completion, retention, assessment completion, missingness, risk assessments, and participant-reported acceptability measures. Group differences in feasibility indicators may be explored using appropriate descriptive or inferential statistics; however, emphasis will remain on estimation, confidence intervals, and interpretation of implementation parameters rather than hypothesis testing.

**Initial Outcome and Descriptive Trend Analyses**. We will first summarize sample characteristics, evaluate baseline comparability between study arms, and examine patterns of missingness. Baseline variables differing across arms and associated with outcomes may be included as prespecified covariates. Primary clinical outcomes will include hazardous drinking and posttraumatic stress symptom severity, consistent with the intervention's principal clinical targets. Exploratory analyses will examine selected transdiagnostic process measures aligned with the intervention model, including emotion regulation difficulties, experiential avoidance, anxiety sensitivity, negative emotionality, coping strategies, posttraumatic maladaptive beliefs, and alcohol craving, to inform future mechanism-focused trials.

Clinical outcomes will be analyzed using mixed-effects models appropriate to outcome distributions (e.g., linear models for approximately normal outcomes; generalized models for count outcomes). Primary analyses will test the time × treatment arm interaction (immediate vs. waitlist) across assessment timepoints using an intention-to-treat framework [142]. Models will include fixed effects for time, treatment arm, and time × arm interactions, relevant covariates, and participant-level random effects. Sensitivity analyses will examine completer-only models and alternative model specifications as appropriate.

Consistent with recommendations for pilot trials, analyses will emphasize estimation rather than efficacy testing. We will report standardized effect size estimates (e.g., Hedges’ *g*) with 95% confidence intervals, descriptive outcome trajectories, and preliminary clinical trends to inform refinement, feasibility assessment, and planning of a future fully powered randomized controlled trial. No interim efficacy analyses are planned.

## 3 Discussion

This protocol addresses an important gap in care for SMW/TGD people by developing and testing a brief, telehealth-delivered, culturally responsive intervention integrating selected UP modules with EW. The study is designed to generate feasibility, acceptability, implementation, and preliminary clinical outcome data to guide refinement of RISE and inform the design of a future fully powered randomized controlled trial. By targeting shared emotional and behavioral processes implicated in hazardous drinking, posttraumatic stress symptoms, and stigma-related distress, RISE aims to provide an integrated approach responsive to the complex clinical and sociocultural contexts affecting SMW/TGD communities. Selected UP modules may help SMW/TGD people examine how alcohol is used to regulate distress, enhance belonging, or access affirmation, while strengthening emotional awareness, cognitive and behavioral flexibility, and alternative strategies for meeting these needs. Emerging work suggests that transdiagnostic UP approaches may reduce posttraumatic stress and alcohol-related symptoms and may be amenable to cultural adaptation and telehealth delivery [75,76,108,109,143]. Other studies note that tailored transdiagnostic treatment approaches, including the UP, that can target numerous co-occurring problems related to underlying emotion regulation difficulties may be a useful approach for trauma-exposed people with co-occurring symptom presentations and ongoing traumatic and stigma-related stressors [143,144]. However, standard UP exposures do not directly target trauma-memory processing or culturally salient stigma-related adversity.

EW may be particularly well suited for integrated interventions targeting hazardous drinking and posttraumatic stress among SMW/TGD populations because writing-based approaches offer flexibility, privacy, autonomy, and opportunities for paced engagement with emotionally salient material, features that may be especially valuable for individuals navigating stigma exposure, disclosure concerns, ambivalence toward trauma-focused treatment, or barriers to affirming care [81,[87], [88], [89], [90], [91], [92], [93],[97], [98], [99], [100], [101], [102], [103], [104], [105]]. In addition, EW permits flexible engagement with traumatic, stigma-related, and identity-related experiences that may not fit neatly within conventional trauma frameworks but remain central to distress and alcohol-related coping among SMW/TGD populations [77]. Accordingly, RISE integrates brief exposure-based EW and population-informed adaptations to support engagement with trauma-, stigma-, and identity-related experiences relevant to SMW/TGD populations.

Several intervention features may support feasibility, accessibility, and clinical relevance, including abbreviated delivery, telehealth implementation, integration of transdiagnostic CBT skills with focused writing-based exposure, and explicit attention to trauma- and stigma-related experiences. Importantly, although several integrated trauma–substance use interventions demonstrate efficacy in general populations, few have been culturally adapted, evaluated, or designed to concurrently address hazardous drinking, posttraumatic stress, and stigma-related distress among SMW/TGD populations. If feasible, acceptable, and supported by evidence from a definitive trial, RISE could represent a scalable approach for addressing hazardous drinking and posttraumatic stress symptoms among SMW/TGD populations and inform future development of integrated behavioral interventions for marginalized communities.

### 3.1 Limitations and future directions

Several limitations should be considered. The pilot design prioritizes feasibility and acceptability rather than definitive efficacy testing; therefore, clinical outcome trends and effect-size estimates should be interpreted cautiously. The waitlist comparator does not adjust for expectancy, attention, or nonspecific treatment effects. Generalizability may also be limited by the regional sampling frame, telehealth access requirements, and inclusion/exclusion criteria.

Future research should first establish clinical effectiveness through a fully powered randomized controlled trial incorporating longer follow-up periods (e.g., 6–12 months) and, where feasible, objective outcome indicators. Subsequent work may evaluate cost-effectiveness, implementation, and scalability in community and healthcare settings. If supported by evidence from a definitive trial, future studies could evaluate the incremental and synergistic effects of individual RISE components, identify mechanisms and moderators of treatment response, and test personalized sequencing strategies to optimize intervention fit and efficiency. Comparative and implementation research could further evaluate RISE relative to treatment as usual, alternative integrated interventions, or lower-intensity approaches (e.g., self-monitoring), while examining scalability, cost-effectiveness, and integration within stepped-care or modular treatment pathways. Within such models, RISE could function as a brief, early-line, adjunctive, or targeted intervention for SMW/TGD individuals requiring integrated support for hazardous drinking, posttraumatic stress, and stigma-related distress.

## 4 Conclusion

This pilot study will evaluate the feasibility, acceptability, and preliminary clinical signals of RISE, a brief intervention integrating selected UP skills with EW to address hazardous drinking and posttraumatic stress symptoms among SMW/TGD populations. Findings will guide refinement of intervention content, cultural adaptations, dosing, supervision procedures, and implementation strategies while informing the design and powering of a future definitive trial. If RISE proves feasible and promising, it may offer a scalable model for integrating transdiagnostic CBT and focused writing-based exposure to address hazardous drinking and posttraumatic stress symptoms among SMW/TGD communities and potentially other underserved populations.

## Ethics approval statement

The current study was approved by the University of Rhode Island (IRB#: 00000599).

## Trial sponsor

Rhode Island Foundation Medical Research Grant (Grant# 155240).

## Protocol version

Version 1.0 (11/22/2025)

## Funding

Jillian Scheer acknowledges support from the 10.13039/100000027National Institute on Alcohol Abuse and Alcoholism under grant K01AA028239. Rachel Girard acknowledges support from the 10.13039/100000026National Institute on Drug Abuse under grant F31DA062435. Emily Helminen acknowledges support from the 10.13039/100000026National Institute on Drug Abuse under grant T32DA016184. The research presented herein is the authors’ own and does not represent the views of the funders, including the National Institutes of Health.

## CRediT authorship contribution statement

**Virinca Jaipuriyar:** Conceptualization, Visualization, Writing – original draft, Writing – review & editing. **Kriti Behari:** Conceptualization, Methodology, Writing – review & editing. **Katya Rashkovsky:** Conceptualization, Methodology, Writing – review & editing. **Rachel Girard:** Conceptualization, Methodology, Writing – review & editing. **Anna L. Sherman:** Conceptualization, Methodology, Writing – review & editing. **Natalie Chasten:** Conceptualization, Methodology, Writing – review & editing. **Gabriella (Gabby) Epshteyn:** Conceptualization, Writing – review & editing. **Joonwoo Lee:** Conceptualization, Writing – review & editing. **Emily C. Helminen:** Conceptualization, Writing – review & editing. **Jillian R. Scheer:** Conceptualization, Funding acquisition, Methodology, Resources, Supervision, Visualization, Writing – original draft, Writing – review & editing.

## Declaration of competing interest

The authors declare that they have no known competing financial interests or personal relationships that could have appeared to influence the work reported in this paper.

## Data Availability

No data was used for the research described in the article.
